# Sublethal herbicide exposure alters cotton susceptibility to aphid infestation

**DOI:** 10.1002/ps.71180

**Published:** 2026-08-11

**Authors:** Purushottam Gyawali, Ubaldo Torres, David L Kerns, Gregory A Sword, Muthukumar V Bagavathiannan

**Affiliations:** ^1^ Department of Soil and Crop Sciences Texas A&M University College Station TX USA; ^2^ Department of Entomology Texas A&M University College Station TX USA

**Keywords:** cotton aphid, herbicide drift, integrated pest management, plant‐mediated herbicide effects, synthetic auxins

## Abstract

**BACKGROUND:**

Herbicides remain vital tools for weed control, yet their effects can extend beyond weeds to affect crop plants, altering plant–insect interactions. Field observations of elevated cotton aphid (*Aphis gossypii*) infestations following a simulated herbicide drift in cotton suggested that sublethal herbicide exposure might increase crop susceptibility to insect pests. To investigate this phenomenon, multiyear field and glasshouse experiments were conducted to determine whether sublethal herbicide exposures, such as those resulting from off‐target drift, modify cotton susceptibility to aphid infestations.

**RESULTS:**

In field studies, sublethal herbicide exposure altered cotton susceptibility to aphids, with herbicide type significantly influencing the extent of infestations. Synthetic auxins (2,4‐d and dicamba) were consistently associated with the highest aphid densities, followed by glufosinate and glyphosate, whereas atrazine, paraquat, isoxaflutole and nicosulfuron suppressed or maintained aphid infestations below those of untreated controls. Aphid severity was highest when applications were made during early vegetative stages and declined as the crop matured. In glasshouse experiments, herbicide effects were strongly dose‐dependent: moderate exposure promoted aphid proliferation, whereas higher doses suppressed aphid populations. Correlations between crop injury from herbicides, relative chlorophyll content and aphid density indicate that herbicide‐associated physiological stress and altered plant vigor influence susceptibility. Spatial analyses revealed higher aphid numbers around auxin‐treated plants, suggesting potential drift‐related or volatile‐mediated influences on aphid behavior or reproduction.

**CONCLUSION:**

This study provides clear evidence that sublethal herbicide exposure to crops can enhance aphid infestation through stress‐induced physiological and ecological mechanisms. By demonstrating consistent, herbicide‐specific patterns across field and glasshouse environments, these results highlight the need to incorporate indirect herbicide effects into integrated pest management and ecological risk assessment frameworks. © 2026 The Author(s). *Pest Management Science* published by John Wiley & Sons Ltd on behalf of Society of Chemical Industry.

## Graphical abstract

Sublethal herbicide exposure altered cotton susceptibility to aphids. Aphid severity was highest when applications occurred at early vegetative stages and declined as the crop matured. In glasshouse, herbicide effects were dose‐dependent.

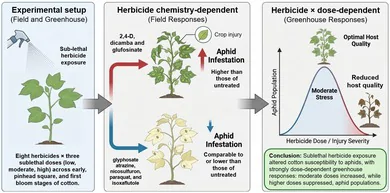

## 1 INTRODUCTION

In agricultural systems, herbicides play a critical role in weed management and maintaining crop productivity.^1^ However, their ecological consequences extend significantly beyond the intended targets, affecting various aspects of the ecosystem,^2^ influencing plant morphology,^3^ physiology,^4^ microbial communities^5^, ^6^ and non‐target organisms,^2^, ^7^, ^8^, ^9^ including herbivore insect pests and beneficial insects.^7^, ^10^, ^11^, ^12^ Additionally, vegetation in field margins, uncultivated lands, and other semi‐natural areas within agricultural landscapes is susceptible to significant ecotoxicological threats from injudicious herbicide applications in adjacent crop fields.^7^, ^9^, ^13^ Although herbicides are primarily designed to suppress weed populations, substantial evidence suggests that some can directly kill insects,^14^ or modulate insect–plant interactions in complex and unexpected ways.^10^, ^15^, ^16^ Understanding these direct and indirect effects is crucial for designing sustainable insect pest and weed management strategies that minimize unintended ecological disruptions and promote long‐term agricultural productivity and ecological balance.

Insect pests are subject to numerous selection pressures that influence habitat changes and predation, which collectively shape their foraging behavior and host selection.^17^, ^18^ These selection pressures range from direct predation and interspecific competition to the physiological constraints imposed by plant chemistry,^17^, ^19^ climate change^20^ and the application of agrochemicals.^21^ In agricultural systems, herbicide applications can alter the ecological landscape,^1^, ^2^, ^9^, ^22^ sometimes contributing to pest outbreaks by disrupting plant–insect dynamics.^4^, ^7^, ^23^, ^24^ These disruptions arise when herbicidal compounds exert unintended toxic effects on key predatory and parasitoid organisms, consequently weakening biological control mechanisms. For instance, a reduction in parasitism rates by the specialist wasp *Aphidius rhopalosiphi* (Hymenoptera: Braconidae) was observed on plants treated with 0.1% of the herbicide 2,6‐dichlorobenzonitrile (DCBN).^25^ Although some herbicides have been associated with direct insect mortality,^14^, ^26^ others have been implicated in enhancing insect fitness, potentially through the suppression of plant defensive mechanisms^27^ or modifying plant volatile emissions^23^, ^28^ that influence herbivores' host‐selection behavior.^15^ Such alterations in plant–insect interactions also may occur when nontarget plants are exposed to sublethal concentrations of herbicides owing to drift.^7^, ^29^, ^30^ These effects are of particular concern in managed agroecosystems, where unintended consequences of herbicide use could alter pest population dynamics, potentially intensifying the need for additional pest control measures.

Aphids (Aphididae) represent one of the most economically significant groups of agricultural pests,^31^, ^32^ known for their rapid reproductive rates and the ability to vector plant pathogens.^33^ Their host selection behavior is driven by a combination of visual and chemical cues, occurring in a sequence of successive events,^34^ which may be influenced by plant stress responses, including those triggered by herbicides.^11^ Although aphid population fluctuations are typically attributed to factors such as temperature,^35^ natural enemies^36^ and plant nutritional status,^37^ an underexplored driver of their infestation patterns is the physiological modification of plants following herbicide exposure.^38^, ^39^ Some herbicides, particularly those that interfere with hormonal pathways,^40^ have been shown to disrupt the levels, activity and crosstalk of key phytohormones such as natural auxins [e.g. indole‐3‐acetic acid (IAA)], jasmonic acid (JA), salicylic acid (SA), ethylene (ET) and abscisic acid (ABA), thereby compromising plant defense mechanisms and increasing susceptibility to herbivorous insects.^10^ However, although previous studies have shown that herbicide exposure can alter plant physiology and subsequent insect responses,^10^, ^11^, ^35^, ^38^, ^39^ relatively few have systematically compared how different herbicide treatments or modes of action (MoA) influence aphid host preference and population dynamics, leaving this relationship poorly understood.

The biochemical mechanisms underlying these interactions involve complex changes in plant secondary metabolites,^41^, ^42^ defense signaling pathways and nutritional composition.^43^, ^44^ Herbicides such as glyphosate and auxin‐mimicking compounds are known to disrupt plant hormonal homeostasis,^4^, ^23^, ^24^, ^39^, ^40^, ^45^ which can indirectly influence insect herbivore preference and performance.^46^ Glyphosate, for example, inhibits the shikimate pathway, affecting the synthesis of essential amino acids and secondary metabolites,^47^ which in turn may alter plant physiology,^4^ and the host's nutritional quality.^48^ Likewise, auxin herbicides can induce abnormal plant growth patterns^49^, ^50^ and reduce defenses, potentially creating more favorable conditions for insect pests.^10^, ^30^ Beyond direct physiological changes, herbicide applications also can influence insect populations by modifying plant community composition^7^ and soil microbial interactions.^5^, ^6^ Many herbicides exhibit residual activity, affecting plant species,^51^ including weeds, that serve as alternate hosts for insect herbivores.^52^ The elimination of specific weed species can lead to shifts in herbivore population dynamics by altering habitat complexity and resource availability.^2^, ^52^, ^53^, ^54^ Additionally, soil microbial communities, which play a crucial role in plant nutrient cycling^55^ and resistance to insect pests,^56^ can be significantly altered by herbicide applications.^22^ These microbial shifts may further contribute to changes in plant chemistry, indirectly affecting herbivore host selection, feeding behavior, and fitness.^57^ The extent to which these herbicide‐induced physiological alterations translate into increased pest susceptibility is an emerging and needed area of research with implications for integrated insect pest and weed management.

During the summer of 2022, while assessing injury to conventional cotton (*Gossypium hirsutum* L.) from simulated herbicide drift, we observed an unexpected but consistent pattern: cotton aphid (*Aphis gossypii* Glover; Hemiptera: Aphididae) infestations and damage were disproportionately higher in plots exposed to certain herbicides at low, sublethal rates intended to mimic off‐target drift. This particular observation raised a critical question: Could sublethal herbicide doses typically associated with drift events inadvertently enhance aphid host‐plant suitability? Based on this observation, we hypothesized that exposure to sublethal doses of certain herbicides, such as those resulting from herbicide drift, would make cotton plants more attractive or susceptible to aphid infestation. This study involved eight different herbicides applied at varying rates across multiple cotton growth stages, providing an opportunity to systematically investigate this phenomenon and test our hypothesis. Beyond observing and rating the severity of aphid infestations, this study also allowed us to assess changes in herbicide‐induced injury patterns and the cumulative effects on aphid infestation. Additionally, using a multi‐environment, multiple‐choice experimental approach, we aimed to determine whether sublethal doses (potential drift) of certain herbicides make cotton plants more attractive to or susceptible to aphid infestations. The results obtained provide important insights into herbicide–arthropod interactions and their implications for ecological understanding and the development of integrated pest management strategies.

## 2 MATERIALS AND METHODS

### 2.1 Field experiments

The initial study evaluating cotton (*G. hirsutum*) response to sublethal herbicide exposure under field conditions was conducted during the summer of 2022 at the Texas A&M University Research Farm near College Station, Texas, USA (30.55° N, −96.43° W). A conventional, herbicide‐susceptible cotton cultivar, NexGen® AM UA 48 (Americot, Inc., Lubbock, TX, USA), was planted at 120 kg ha^−1^ on 2 May 2022 following uniform field preparation, with crop management practices guided by Texas A&M AgriLife Extension recommendations for cotton production in the region.^58^ To assess growth‐stage‐specific responses to sublethal herbicide exposure, the field was divided into three spatially discrete, adjacent experimental areas, each assigned to herbicide application at a distinct developmental stage: early vegetative (6–7 leaves), pinhead square and first bloom. Because crop developmental stage was inherently linked with application timing, each growth stage was analyzed as a separate stage‐specific experiment. This approach was used to assess herbicide treatment effects within each developmental stage rather than to make direct statistical comparisons among stages, which could be confounded by differences in application date, crop phenology and field area.

Within each growth‐stage experiment, treatments were arranged in a full factorial design consisting of eight herbicides, an untreated control, and three sublethal application rates (low, moderate and high). Sublethal rates were predetermined to induce ≈50–60% visible foliar injury, a level commonly associated with a realistic off‐target herbicide drift scenario. Herbicide identities and corresponding rates are provided in Table 1. Each experiment followed a randomized complete block design (RCBD) with four replications, and all 27 treatment combinations (9 treatments × 3 rates) were randomized independently within each replication.

**Table 1:** Herbicides and doses applied in both field and glasshouse experiments

Trade name	Active ingredient and manufacturer	Full rate (1X)	Sublethal doses used		
			Low	Moderate	High
		**g ai or ae ha** ^**−1**^			
Enlist One®	2,4‐Dichlorophenoxyacetic acid; Corteva Agriscience, Indianapolis, IN, USA	≈1065	11 (0.01X)	53 (0.05X)	106 (0.10X)
Atrazine 4 L®	Atrazine; Syngenta, Basel, Switzerland	≈2242	560 (0.25X)	1121 (0.50X)	1681 (0.75X)
XtendiMax®	Dicamba; Bayer CropScience, St Louis, MO, USA	≈560	28 (0.05X)	67 (0.12X)	112 (0.2X)
Liberty®	glufosinate–ammonium; Bayer CropScience	≈594	74 (0.125X)	149 (0.25X)	297 (0.50X)
Roundup PowerMaxx®	Glyphosate; Monsanto Company, St Louis, MO, USA	≈1262	158 (0.125X)	315 (0.25X)	630 (0.50X)
Alite 27®	Isoxaflutole; Bayer CropScience	≈105	26 (0.25X)	53 (0.50X)	74 (0.70X)
Accent Q®	Nicosulfuron; Syngenta	≈34.4	15 (0.44X)	23 (0.66X)	30 (0.88X)
Gramoxone®	Paraquat dichloride; Syngenta	≈560	70 (0.125X)	140 (0.50X)	280 (0.50X)

*Note:* Values in parentheses denote the X rate, representing the fraction of the full recommended herbicide rate (g ai or ae ha^−1^).

Each plot consisted of four rows spaced 1.0 m apart and 7.6 m in length, resulting in a treated plot area of approximately 30.4 m^2^. A 1.5‐m alley separated plots to minimize physical and spray interference among treatments. Herbicides were applied only to the two center rows of each plot using a CO_2_‐pressurized backpack sprayer equipped with a four‐nozzle boom. The sprayer was calibrated to deliver 140 L ha^−1^ at a ground speed of 1.33 m s^−1^. To minimize herbicide drift, synthetic auxin herbicides (2,4‐d and dicamba) were applied using TTI11002 (TurboTeeJet® Induction) nozzles (Spraying Systems Co., Wheaton, IL, USA), which generate coarse droplets, whereas all other herbicides were applied using TT11002 (TurboTeeJet®) nozzles (Spraying Systems Co.). Applications were made at wind speeds <10 km h^−1^, air temperatures between 25 and 30 °C, and relative humidity (RH) 60–70%.

In order to validate and confirm the findings from the 2022 field observations, a follow‐up field study was conducted in 2023 using a simplified approach. The same cotton cultivar was planted on 5 May 2023, and the experiment was conducted as an RCBD with four replications. Based on the 2022 results, which indicate that early‐season herbicide exposure was most strongly associated with subsequent aphid infestation and that aphid responses were not significantly influenced by herbicide dose, only the early vegetative (6–7 leaves) growth stage and a single moderate sublethal herbicide rate (Table 1), along with an untreated control, were evaluated. Consequently, the 2023 study focused on a single, representative exposure scenario to evaluate the consistency and reproducibility of herbicide‐aphid interactions.

In both years, to eliminate confounding effects from weed competition, plots were maintained weed‐free throughout the growing season using a uniform pre‐emergence application of acetochlor (Warrant®; Bayer CropScience, St Louis, MO, USA) at 1.68 kg ai ha^−1^, followed by periodic manual hoeing and intrarow cultivation, as needed. The same pre‐emergence herbicide and rate were applied uniformly across all experimental units to ensure consistency and avoid confounding effects. No supplemental fertilizer was applied during the field experiments to avoid confounding herbicide injury responses with nutrient‐induced differences in plant vigor. All treatments, therefore, experienced similar baseline soil fertility.

### 2.2 Glasshouse experiments

#### 2.2.1 Insect and plant Materials

The cotton aphid (*A. gossypii*) colony used in this study was sourced from the Sword Lab at the Department of Entomology, Texas A&M University, College Station, TX, USA. The colony was maintained under laboratory conditions at 25 ± 1 °C, 70 ± 10% RH and a 14 h:10 h, light:dark photoperiod on the same cotton variety used in field experiments. Plants were grown in a controlled growth chamber maintained at 27 ± 1 °C, 70 ± 10% RH and a 14 h:10 h, light:dark photoperiod. Likewise, all experimental cotton plants were grown in a controlled growth chamber at the Borlaug Center for Southern Crop Improvement, Texas A&M University, College Station, TX, USA. Plants were cultivated in plastic pots (12 cm tall × 10 cm diameter) filled with Jolly Gardener® Pro‐Line C/20 commercial growing media (Oldcastle Lawn & Garden; Atlanta, GA, USA) and maintained under 27 ± 1 °C, 70 ± 10% RH and a 14 h:10 h, light:dark photoperiod until they reached the early vegetative growth stage (6–7 leaves) for treatments. Plants were watered daily and uniformly fertilized at the manufacturer‐recommended concentration every 2 weeks with Miracle‐Gro® All Purpose Soluble Plant Food (Scotts Miracle‐Gro Company, Marysville, OH, USA).

#### 2.2.2 Multiple‐choice experiment

A multiple‐choice experiment was conducted in 2024 under controlled glasshouse conditions (26 ± 1 °C, 70 ± 10% RH and a 14 h:10 h, light:dark photoperiod) to evaluate the preference of *A. gossypii* for cotton plants treated with various sublethal doses (low, moderate and high; Table 1) of different herbicides. The study followed an RCBD design, and the methodology was adapted with slight modifications from previous studies that used multiple‐choice experiments to assess insect preference, performance and host‐selection behaviors.^59^, ^60^ The graphical representation of the experimental setup used in this study for the multiple‐choice experiment is presented in Fig. 1.

**Figure 1:**
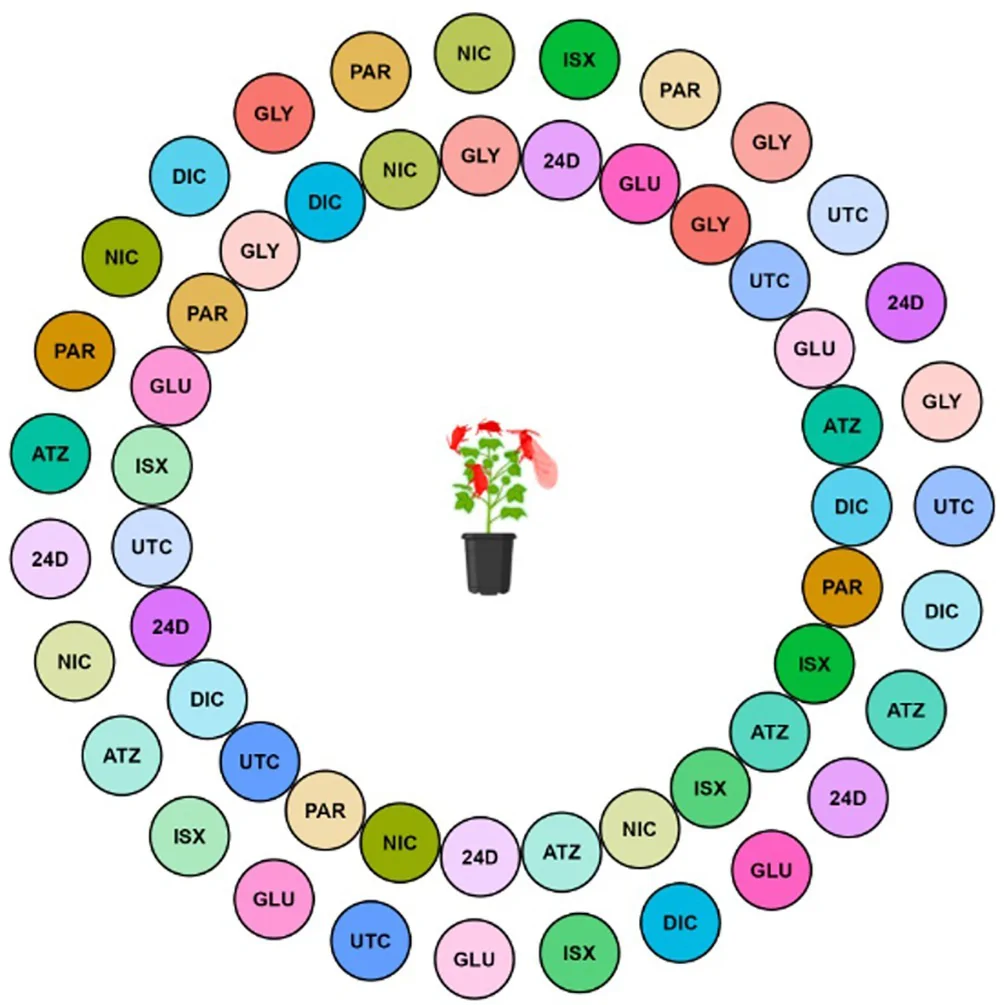
Experimental setup for multiple‐choice experiments regarding aphid host selection. A potted cotton plant at the early vegetative growth stage (6–7 leaves), infested with aphids and placed in the center serves as the aphid source. Two concentric rings of potted cotton plants surrounded the source, each representing a single replicate pot. Plants received one of eight herbicide treatments (24D = 2,4‐D; ATZ, atrazine; DIC, dicamba; GLU, glufosinate; GLY, glyphosate; ISX, isoxaflutole; NIC, nicosulfuron; PAR, paraquat; and UTC, untreated control) at one of three doses (high, moderate and low). Herbicide type is indicated by color and dose is indicated by transparency (darker = high, lighter = low).

Once the potted cotton plants reached the 6–7 leaf stage, they were labeled with unique colors and numerical codes to ensure proper identification of treatments. Eight herbicides were applied at three sublethal doses (Table 1) using a calibrated spray chamber (DeVries Manufacturing Inc, Hollandale, MN, USA) with a delivery rate (140 L ha^−1^) consistent with the application rates used in the field experiments. Following herbicide application, treated plants were placed outdoors for 2 days to facilitate herbicide absorption, simulate field conditions and mitigate the potential drift effects of auxin herbicides on other treatment groups. Untreated (control) plants were not sprayed but also were placed outdoors alongside the treated plants.

The plants were arranged equidistantly in two concentric circles with a radius of 1.0 m (inner circle) and 1.5 m (outer circle), with one potted plant per treatment (herbicide × dose) placed in each ring on the glasshouse floor. This arrangement was replicated three times, resulting in three separate circles, each consisting of two concentric rings (Fig. 1). A cotton plant heavily infested with laboratory‐reared *A. gossypii* was then placed at the center of each concentric circle (Fig. 1), allowing aphids to disperse freely and select their preferred host plants for settlement and reproduction. Aphid counts, herbicide injury rating, and Soil Plant Analysis Development (SPAD) readings were recorded 30 days after aphid release.

### 2.3 Data collection

#### 2.3.1 Field experiment

In 2022, visual assessments of aphid infestation and herbicide‐induced injury were initiated immediately after elevated aphid densities were first observed in herbicide‐treated plots [≈39 days after treatment (DAT) at the early vegetative stage, 23 DAT at the pinhead square stage and 7 DAT at the first bloom stage]. Aphid infestation was visually rated on a standardized 0–10 scale (Table 2), where 0 indicated no infestation, and each increment corresponded to greater infestation severity. A rating of 5 indicated that ≈50% of the plants within the treated plot exhibited severe aphid damage, whereas a rating of 10 represented nearly complete infestation, where aphids densely colonized all plants, with widespread honeydew accumulation and sooty mold development on leaves and the ground beneath the plants (Table 2; Fig. 2); honeydew accumulation is a characteristic indicator of intense aphid‐feeding activity. Herbicide‐induced cotton injury was estimated from visible foliar damage, including chlorosis, necrosis, twisting, curling and stunting, with symptom characterization corresponding to the typical injury patterns associated with each herbicide treatment.

**Table 2:** Key to visual rating of aphid damage on herbicide‐treated cotton

Scale	Aphid damage description
0	No aphids or damage observed.
1	A few aphids on isolated plants; no visible damage.
2	Low aphid infestation on scattered plants with minimal visible leaf damage.
3	Light aphid presence on multiple plants with slight leaf curling or yellowing.
4	Moderate aphid infestation with noticeable leaf curling and slight honeydew presence.
5	Heavy aphid presence on leaves with moderate leaf distortion, and honeydew accumulation is visible on >50% of the plants.
6	Severe aphid infestation with extensive leaf curling and yellowing, with honeydew coating present on >60% of the plants.
7	Very heavy aphid infestation with a significant leaf distortion, yellowing, and honeydew buildup with sooty mold development on most of the plants.
8	Extensive aphid damage on >80% of plants with severe leaf curling and yellowing, heavy honeydew and sooty mold affecting plant growth.
9	Near‐total plant coverage (>90%) with aphids; severe leaf damage, widespread sooty mold, significant reduction in plant vigor and growth.
10	Complete aphid colonization with plant functions highly impaired, extensive leaf curling, heavy sooty mold and significant growth suppression.

**Figure 2:**
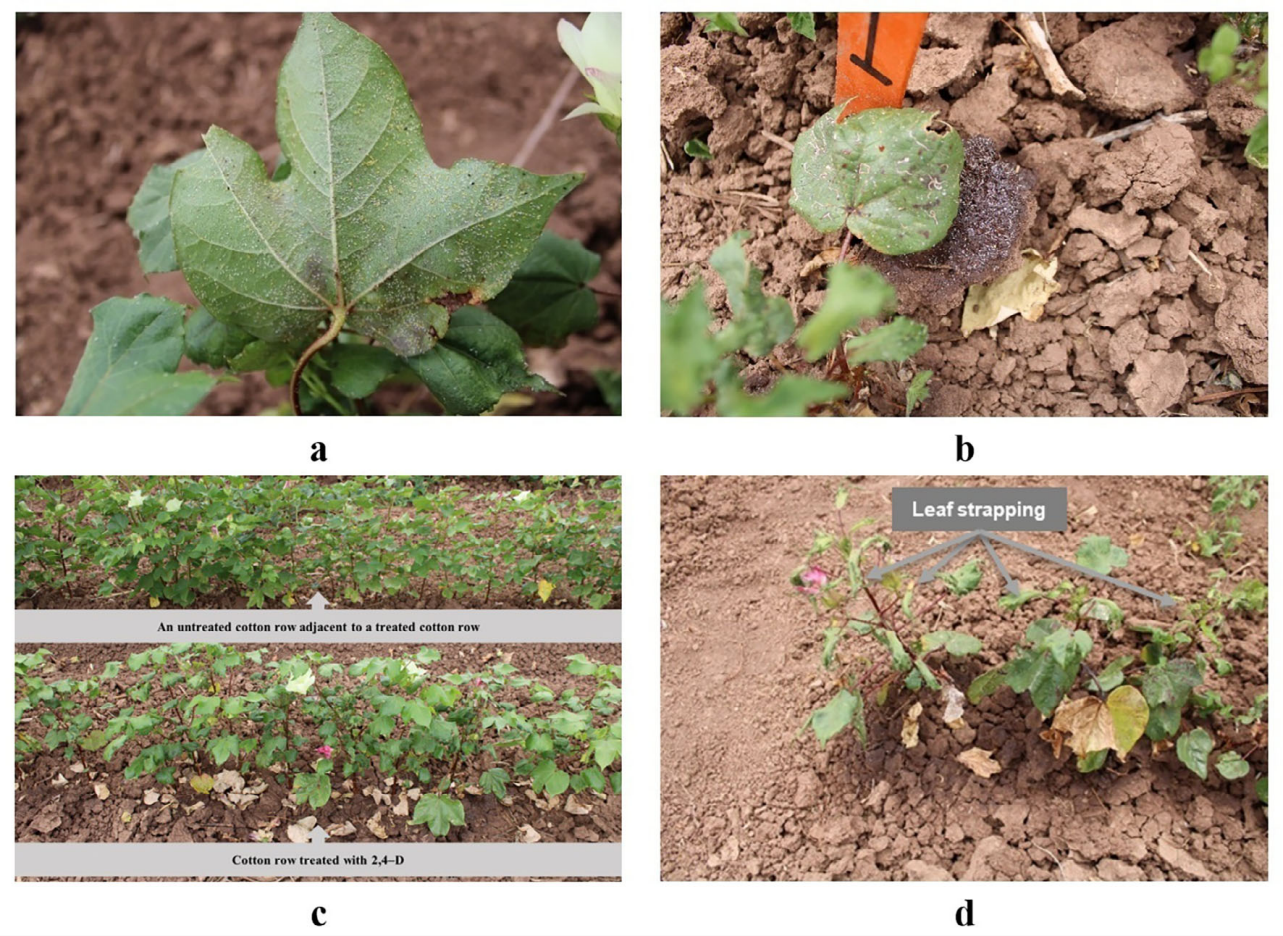
Cotton plants exhibiting severe auxinic herbicide (2,4‐d) injury and aphid infestation. (a) Cotton leaf heavily infested with aphids; (b) honeydew accumulation on the soil beneath an aphid‐infested leaf; (c) cotton row treated with 2,4‐D showing extensive aphid infestation and crop injury compared with an adjacent untreated row; and (d) cotton plants exhibiting characteristic 2,4‐d injury symptoms (including leaf strapping, epinasty and plant stunting) combined with severe aphid damage.

The same rating scale was used for the field study conducted in 2023; however, aphid infestation was assessed weekly for 3 weeks beginning 7 days after the initial infestation was observed, which occurred ≈30 DAT. Accordingly, assessments were conducted at 37, 44 and 51 days after herbicide application. Additionally, plant greenness was assessed in 2023 using SPAD values measured with the *SPAD 502 Plus* Chlorophyll Meter (originally developed by Konica Minolta, now distributed by Spectrum Technologies, Inc., Aurora, IL, USA), providing a quantitative measure of chlorophyll content. SPAD readings were collected from six randomly selected cotton plants per plot, excluding the oldest basal leaves, with values averaged from three leaves per plant sampled from the upper two‐thirds of the canopy.

#### 2.3.2 Glasshouse experiment

In the glasshouse experiment, aphid abundance was quantified by recording counts on individual plants across all treatment groups, rather than relying on visual infestation ratings, providing a more precise quantification of infestation levels. The plant greenness index (SPAD values) was obtained by measuring a minimum of three leaves per plant selected from the upper two‐thirds of the canopy. Herbicide‐induced injury was visually assessed for each plant as described previously.

### 2.4 Statistical analysis

All statistical analyses were performed in R statistical software v4.4.3^61^ using generalized linear mixed models (GLMMs) implemented in the glmmtmb package.^62^ For the 2022 field experiment, data were analyzed separately for each cotton growth stage, with herbicide, dose and their interaction considered as fixed effects, and replication as a random effect. Aphid severity ratings were modeled using a beta distribution with a logit link. For the 2023 field experiment, analyses were conducted separately for each observation week; each weekly dataset was analyzed using the same model structure (herbicide as a fixed effect; replication as a random effect) and the same beta‐distributed response with a logit link. For the 2024 glasshouse experiment, aphid counts per plant were modeled using a Poisson GLMM, with herbicide and dose as fixed effects and replication as a random effect. Model diagnostics, including checks for overdispersion and residual uniformity, were performed using the DHARMa package.^63^ No evidence of overdispersion or residual nonuniformity was detected; therefore, the fitted models were retained for inference.

For all analyses, the significance of fixed effects was evaluated using Type II Wald chi‐square tests (analysis of deviance). When significant effects were detected, estimated marginal means (EMMs; equivalent to least‐squares means) were computed using the emmeans package^64^ and back‐transformed to the response scale for interpretation. Pairwise comparisons among treatment means were performed using Fisher's least significant difference (LSD) at α = 0.05. Statistically distinct means were identified using compact letter displays (CLDs) generated with the multcomp package,^65^ and graphics were produced using ggplot2.^66^ Treatment differences were summarized both numerically and graphically.

Pairwise correlations among key agronomic and pest‐related variables, including herbicide dose, crop growth stage, crop injury rating, SPAD and aphid infestation, were examined separately for each herbicide treatment within each study year. Because several variables were non‐normally distributed or ordinal in nature (for example, dose levels and growth stages), Spearman's rank correlation coefficients (ρ) were used to quantify the strength and direction of associations. Statistical significance was evaluated using two‐tailed tests, with thresholds set at *P* < 0.05 (*), P < 0.01 (**) and *P* < 0.001 (***); nonsignificant associations denoted as ‘ns” (*P* ≥ 0.05).

## 3 RESULTS

### 3.1 Field experiment 2022

Aphid infestation severity in the 2022 field experiment varied according to herbicide and cotton growth stage at the time of exposure. When analyzed separately by growth stage, herbicide had a highly significant effect on aphid severity during the early vegetative stage (χ^2^_8_ = 51.11, *P* < 0.0001) and the pinhead square stage (χ^2^_8_ = 27.77, *P* < 0.001), whereas no significant effect was detected at the first flower stage (*P* > 0.05). Herbicide dose did not significantly affect aphid severity during the early vegetative (*P* > 0.05), but a significant dose effect was observed at the pinhead square stage (χ^2^_2_ = 6.14, *P* = 0.046). No herbicide × dose interactions were detected at any growth stage (*P* > 0.05). Aphid infestation responses to herbicide treatments across growth stages, along with herbicide‐induced crop injury (represented by point size), are shown in Fig. 3.

**Figure 3:**
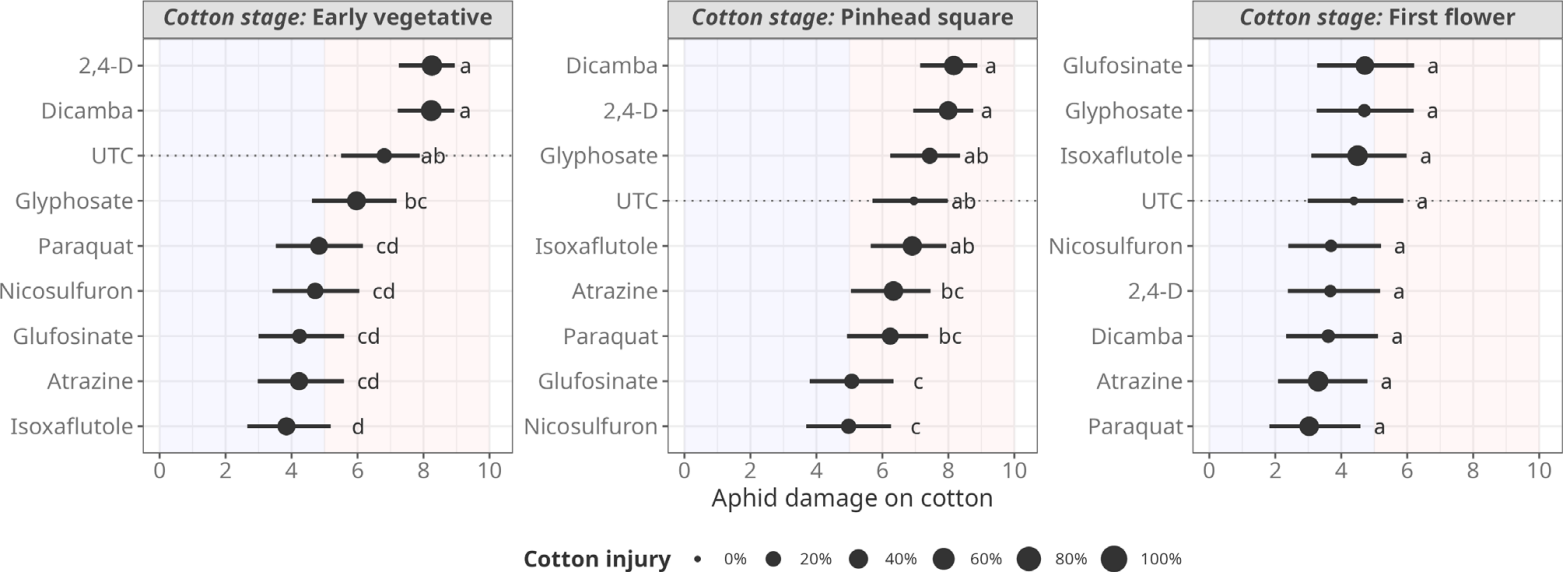
Mean (± SE) aphid damage severity on a 0–10 scale (0 = no aphids observed, 10 = highest infestation observed in the trial) on cotton as influenced by herbicide across three crop growth stages under field conditions in 2022. Assessments were conducted ≈39 DAT at the early vegetative stage, 23 DAT at the pinhead square stage and 7 DAT at the first flower stage. Panels represent herbicide applications made at the early vegetative, pinhead square and first flower growth stages. Points indicate mean aphid damage per plot, with horizontal bars representing standard errors. Circle size denotes the associated level of cotton injury (%) for each herbicide, as shown in the legend. Different lowercase letters within a panel indicate significant differences among herbicide treatments based on Fisher's LSD test at α = 0.05. UTC,  untreated control.

At the early vegetative stage, aphid damage was greatest in plots treated with the synthetic auxin herbicides 2,4‐d and dicamba, which did not differ from each other but resulted in significantly higher aphid severity than the untreated control (UTC) and all other herbicide treatments (Fig. 3). Glyphosate exposure produced moderate levels of infestation, whereas atrazine, paraquat, nicosulfuron, glufosinate and isoxaflutole were associated with significantly lower aphid severity, with isoxaflutole resulting in the lowest infestation overall. At the pinhead square stage, aphid infestation severity remained highest following exposure to dicamba and 2,4‐d, although differences among herbicides were less pronounced than at the early vegetative stage. Glyphosate, the UTC and isoxaflutole resulted in intermediate aphid responses, whereas atrazine and paraquat produced lower infestation levels. Glufosinate and nicosulfuron were associated with the lowest aphid severity at this stage. Although a significant dose effect was detected, the absence of herbicide × dose interaction indicates that relative differences among herbicides were consistent across dose levels. Aphid damage was slightly lower at the moderate dose than at the low or high dose, indicating a nonlinear dose–response pattern, which was most likely to be a consequence of natural variations. By the first flower stage, herbicide effects had dissipated; no statistical differences were detected among treatments and aphid injury levels were uniformly low.

### 3.2 Field experiment 2023

Analysis of deviance from beta‐regression GLMMs revealed significant herbicide effects at 37 DAT (χ^2^_8_ = 48.29, *P* < 0.0001), 44 DAT (χ^2^_8_ = 41.97, *P* < 0.0001) and 51 DAT (χ^2^_8_ = 21.12, *P* = 0.007), confirming that herbicide consistently affected aphid infestation, though treatment differences declined over time (Fig. 4). At 37 DAT, infestation was highest in the 2,4‐D, dicamba and glufosinate treatments, followed by glyphosate, whereas atrazine, paraquat, nicosulfuron and isoxaflutole had the lowest aphid ratings. At 44 DAT, overall aphid severity decreased, but glufosinate, 2,4‐d and dicamba remained significantly higher than other treatments. By 51 DAT, the influence of herbicide treatments on aphid severity diminished further, with most herbicides showing overlapping mean separations. Although glufosinate, dicamba and 2,4‐d continued to show relatively higher infestation levels, differences among treatments were less distinct (Fig. 4).

**Figure 4:**
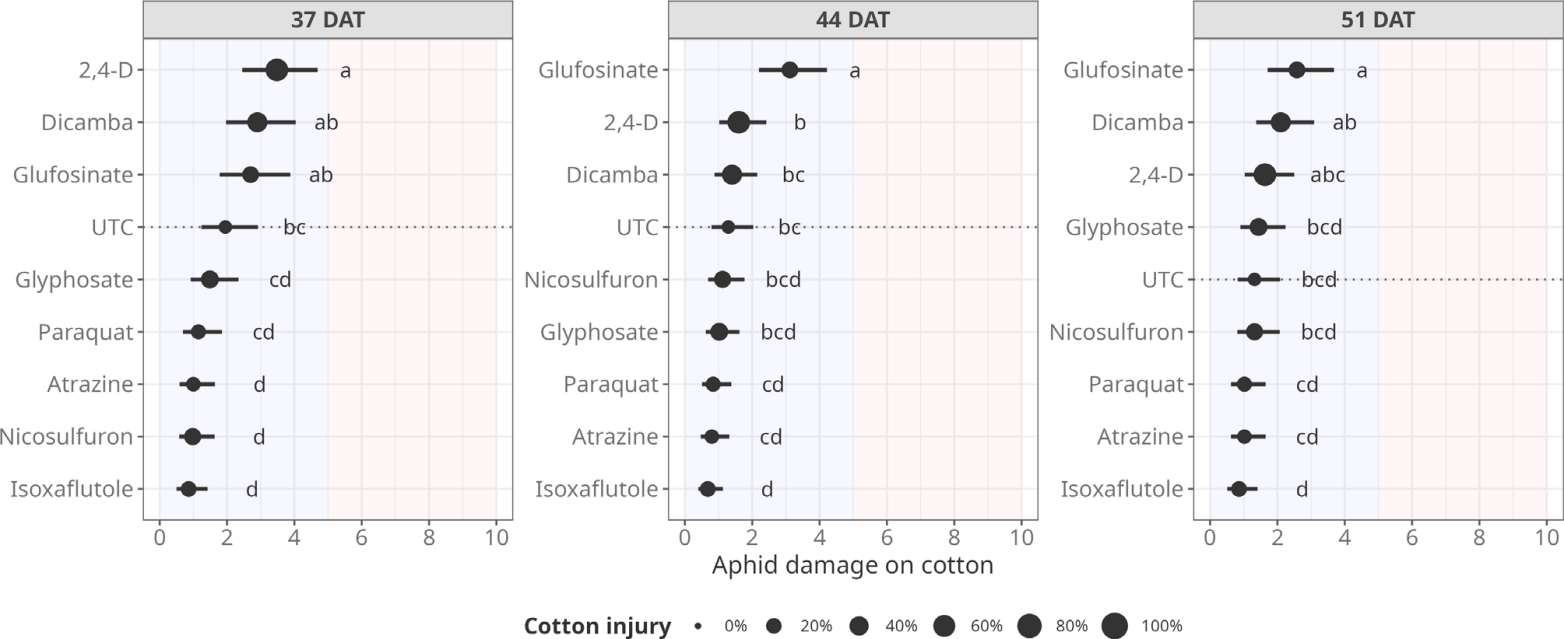
Mean (± SE) aphid damage severity on a 0–10 scale (0 = no aphids observed, 10 = highest infestation observed in the trial) on cotton as influenced by herbicide and DAT in the 2023 field experiment. All herbicide applications were made at the early vegetative growth stage. Panels represent evaluations conducted at 37, 44 and 51 DAT. Each point indicates the mean aphid damage per plot, with horizontal bars showing standard errors. Circle size denotes the level of cotton injury (%) associated with each herbicide, as shown in the legend below the panels. Different lowercase letters within a panel indicate statistically significant differences among herbicide treatments based on Fisher's LSD at α = 0.05. UTC, untreated control.

### 3.3 Glasshouse experiment

The results from the glasshouse experiment revealed a strong herbicide × dose interaction (χ^2^_15_ = 12 455.6, *P* < 0.0001), indicating that the effect of each herbicide on aphid infestation depended on dose level (Fig. 5). At the low dose, aphid counts were highest in plants treated with glufosinate, followed by paraquat, nicosulfuron and 2,4‐d, all of which exhibited significantly greater infestations than the UTC. By contrast, dicamba and atrazine treatments produced the lowest aphid densities. At the moderate dose, dicamba and nicosulfuron supported the highest aphid counts, statistically exceeding the control and most other herbicides, whereas atrazine and 2,4‐d showed the lowest infestation levels. Interestingly, glufosinate, which caused the highest infestation at low dose, exhibited intermediate aphid levels at the moderate dose, suggesting a nonlinear response pattern potentially associated with sublethal plant stress thresholds. At the high dose, the pattern shifted substantially: glyphosate resulted in the greatest aphid infestation, followed by atrazine, whereas paraquat and dicamba produced the lowest aphid counts (Fig. 5).

**Figure 5:**
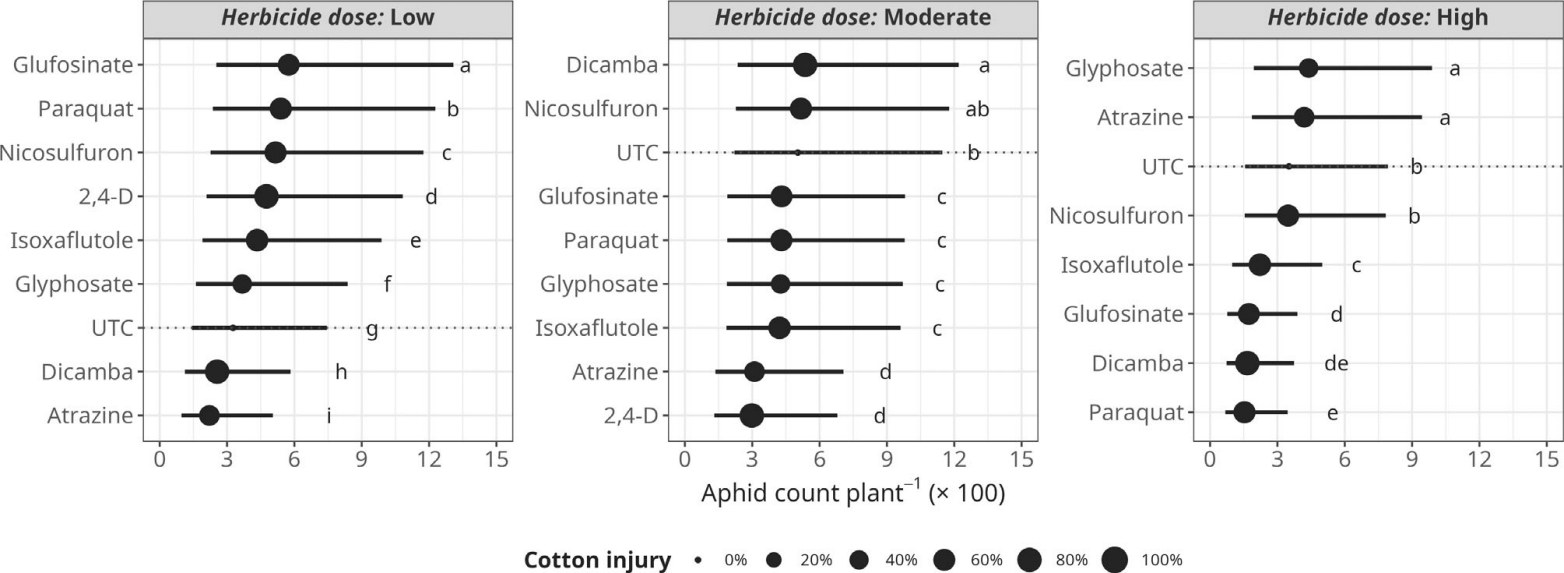
Mean (±SE) aphid counts per cotton plant as influenced by herbicide chemistry and dose under glasshouse conditions (2024). All herbicide applications were made at the early vegetative growth stage, and measurements were taken ≈30 DAT. Panels represent low, moderate and high herbicide doses. Each point indicates the mean aphid density, with horizontal bars showing standard error. Circle size denotes the percentage of cotton injury recorded for each herbicide (legend below the panels). Different lowercase letters within each panel indicate statistically significant differences among herbicide treatments based on Fisher's LSD at α = 0.05. UTC, untreated control.

Across all experiments, aphid infestation consistently varied with herbicide and environmental context. In both field studies, synthetic auxins (2,4‐d and dicamba) and glufosinate increased aphid infestation, whereas treatments with atrazine, paraquat, isoxaflutole, and nicosulfuron reduced aphid infestation. By contrast, the glasshouse study revealed a dose‐dependent reversal: moderate herbicide stress increased aphid infestation, whereas high doses suppressed it, potentially as a result of substantial plant injury. Across these environments and years, isoxaflutole was consistently associated with aphid infestations that were significantly lower than those of the untreated control.

### 3.4 Spatial distribution of aphid infestation in response to herbicides

In order to illustrate the effects of sublethal herbicide exposure on aphid infestation, heat maps were generated from mean aphid severity ratings in the 2022 field study and mean aphid counts in the 2024 glasshouse experiment, visualizing spatial patterns of infestation under both field and glasshouse conditions. Spatial heat maps from the 2022 field experiment revealed distinct herbicide‐ and growth stage‐dependent clustering of aphid infestation (Fig. 6). Across all stages, plots treated with synthetic auxin herbicides, dicamba and 2,4‐D, consistently exhibited the highest aphid damage. Infestation hotspots were not confined to treated plots; aphid pressure extended into adjacent and downwind areas, forming gradients of decreasing abundance. This spillover was most evident during the second growth stage, when auxin‐treated plots acted as focal points of colonization surrounded by zones of moderate aphid activity. By the third growth stage, overall densities declined and aggregation patterns became more diffuse; however, dicamba and 2,4‐d plots remained persistent hotspots, suggesting that these plants remained more suitable as hosts at later crop stages. This pattern was similar in the 2023 field trial as well.

**Figure 6:**
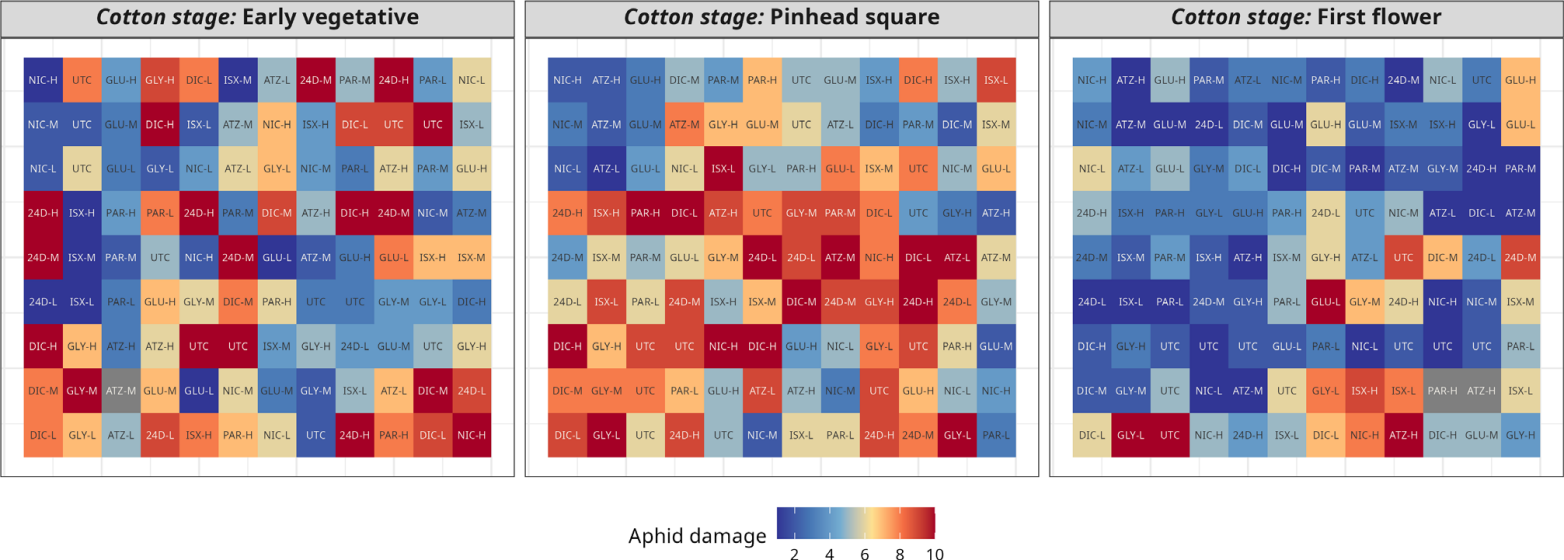
Spatial distribution of aphid infestation across herbicide treatments in the 2022 field trial. Heatmaps depict mean aphid infestation ratings on a 0–10 scale (0 = no aphids observed, 10 = highest infestation observed in the trial) across individual field plots. Each colored cell represents a single plot in its actual field position and corresponds to a specific herbicide × dose × replication combination. The three panels represent herbicide applications made at (a) early vegetative, (b) pinhead square, and (c) first bloom growth stages. Multiple occurrences of a given treatment code within each panel reflect plot replication. Treatment codes are: 24D, 2,4‐d; ATZ, atrazine; DIC, dicamba; GLU, glufosinate; GLY, glyphosate; ISX, isoxaflutole; NIC, nicosulfuron; PAR, paraquat; and UTC, untreated control. Letters following each treatment code indicate herbicide dose: L, low; M, moderate; and H, high.

A comparable spatial pattern was observed in the 2024 glasshouse experiment (Fig. 7). Aphid infestation severity increased along the structural axis of the facility, with lower infestations near the entrance [Fig. 7(c)] and higher densities toward the interior [Fig. 7(a), (b)]. Within high‐density areas, plants near glufosinate‐ and synthetic auxin‐treated pots exhibited elevated aphid counts, even in neighboring untreated replicates, indicating proximity‐dependent amplification.

**Figure 7:**
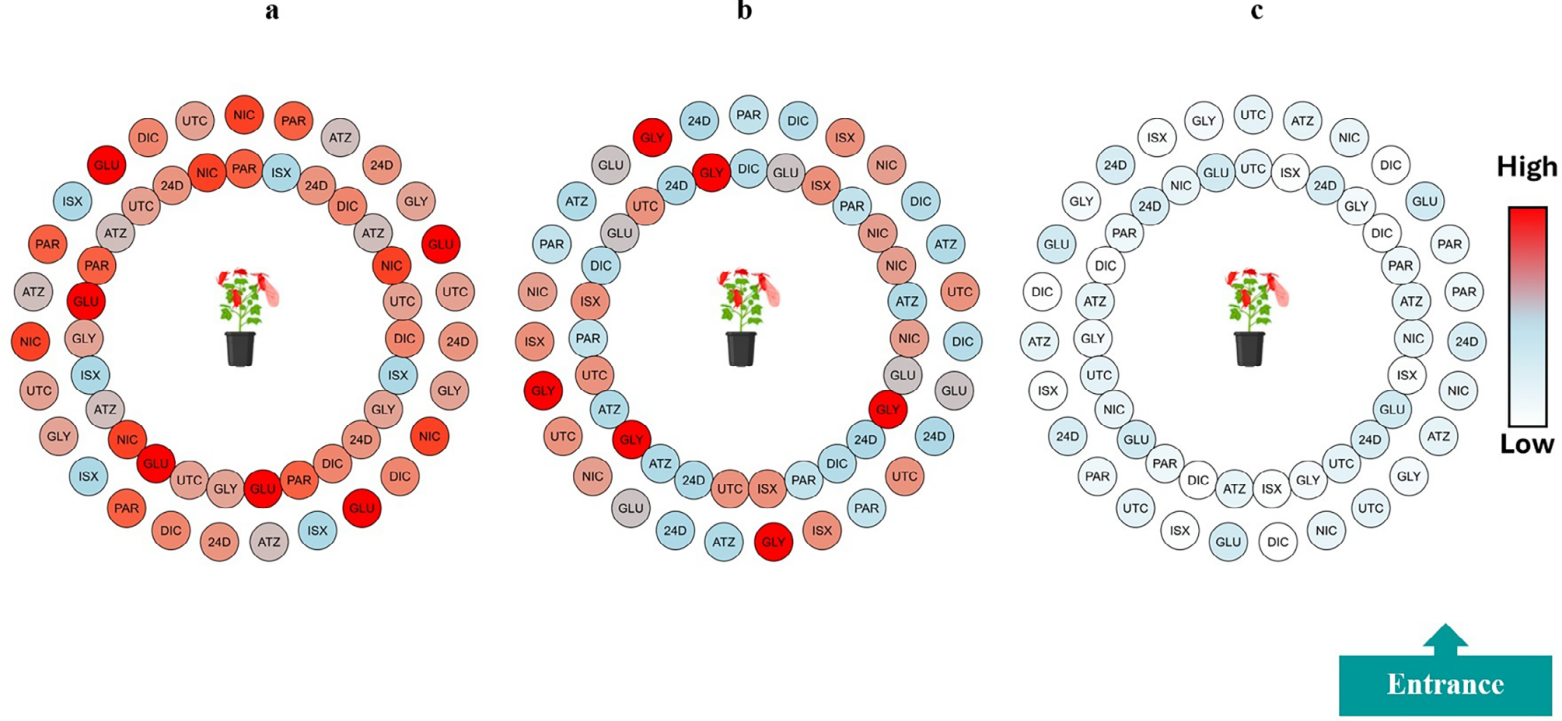
Spatial distribution of aphid counts across herbicide‐treated plants in the glasshouse assay (2024). Heatmaps show aphid counts per plant across three replicates, each containing a randomized distribution of herbicide treatments and their doses. (a, b and c) represent the three experimental replicates, with plants arranged according to their physical positions in the glasshouse. Treatment codes within each circle correspond to specific herbicides: 24D, 2,4‐d; ATZ, atrazine; DIC, dicamba; GLU, glufosinate; GLY, glyphosate; ISX, isoxaflutole; NIC, nicosulfuron; PAR, paraquat; and UTC, untreated control. The ‘Entrance’ label denotes the single glasshouse access door, indicating the physical entry point relative to the plant layout.

### 3.5 Links between crop stage, herbicide injury, and aphid severity

Across studies, crop injury showed moderate to strong correlations with both SPAD values and aphid infestation. However, the direction and magnitude of these correlations varied by herbicide (Table 3). The synthetic auxin herbicides 2,4‐d and dicamba often showed positive correlations between crop injury and aphid infestation, whereas other herbicides, such as glufosinate and paraquat, showed negative or inconsistent associations across years. The growth stage at application was consistently and inversely correlated with crop injury in the 2022 field trial, suggesting that earlier applications increased phytotoxicity. In the glasshouse (2024), increasing herbicide dose was frequently associated with reduced SPAD and aphid levels, particularly under glufosinate and paraquat. Notably, SPAD–aphid relationships also showed negative trends in some treatments. These trends were especially pronounced in the 2023 field study, where SPAD and aphid ratings exhibited strong inverse correlations under glyphosate, glufosinate, atrazine and 2,4‐d, indicating a potential threshold relationship between plant greenness and susceptibility. Injury–SPAD correlations in 2023 also varied widely in direction and strength, ranging from moderately positive (e.g. glyphosate) to strongly negative (e.g. atrazine, paraquat and nicosulfuron), further underscoring herbicide‐specific physiological effects. Although several herbicides, such as isoxaflutole, showed minimal or inconsistent correlations, others, such as atrazine, displayed reversals in directionality across years and parameters.

**Table 3:** Correlations among plant traits (injury, SPAD, growth stage) and aphid severity/count across herbicide treatments

Correlation parameters	24D	ATZ	DIC	GLU	GLY	ISX	NIC	PAR	UTC
**2022‐Field**									
Herbicide dose × crop injury	0.22 ^ns^	0.28 ^ns^	0.2 ^ns^	0.24 ^ns^	0.25 ^ns^	0.26 ^ns^	0.33 ^ns^	0.31 ^ns^	0 ^ns^
Herbicide dose × aphid damage	0.27 ^ns^	−0.07 ^ns^	−0.05 ^ns^	−0.01 ^ns^	−0.1 ^ns^	−0.1 ^ns^	0.08 ^ns^	0.18 ^ns^	0.06 ^ns^
Growth stage × crop injury	−0.77 ***	0.7 ***	−0.74 ***	0.62 ***	−0.83 ***	0.65 ***	−0.69 ***	0.3 ^ns^	−0.54 ***
Growth stage × aphid damage	−0.6 ***	−0.33 ^ns^	−0.64 ***	0.09 ^ns^	−0.21 ^ns^	0.09 ^ns^	−0.1 ^ns^	−0.33 ^ns^	−0.31 ^ns^
Crop injury × aphid damage	0.59 ***	−0.39 *	0.55 ***	−0.11 ^ns^	0.25 ^ns^	0.15 ^ns^	−0.02 ^ns^	−0.4 *	−0.03 ^ns^
**2023‐Field**									
Crop injury × SPAD	0.32 ^ns^	−1 ***	0.74 ^ns^	−0.32 ^ns^	0.63 ^ns^	−0.63 ^ns^	−0.6 ^ns^	−0.63 ^ns^	−0.95 ^ns^
Crop injury × aphid damage	−0.5 ^ns^	0.77 ^ns^	−0.83 ^ns^	−0.32 ^ns^	0.83 ^ns^	‐	−0.63 ^ns^	0.82 ^ns^	0.82 ^ns^
SPAD × aphid damage	−0.95 ^ns^	−0.77 ^ns^	−0.32 ^ns^	−0.8 ^ns^	0.95 ^ns^	‐	−0.21 ^ns^	−0.26 ^ns^	−0.77 ^ns^
**2024‐Glasshouse**									
Herbicide dose × SPAD	−0.26 ^ns^	−0.05 ^ns^	−0.06 ^ns^	−0.65 *	0.44 ^ns^	−0.29 ^ns^	−0.38 ^ns^	−0.67 *	‐
Herbicide dose × aphid counts	−0.26 ^ns^	0.28 ^ns^	0.14 ^ns^	−0.48 ^ns^	−0.09 ^ns^	−0.13 ^ns^	−0.01 ^ns^	−0.07 ^ns^	‐
Herbicide dose × crop injury	0.48 ^ns^	0.51 ^ns^	0.59 *	0.78 **	0.51 ^ns^	0.70 **	0.32 ^ns^	0.63 *	‐
SPAD × aphid counts	0.03 ^ns^	−0.50 ^ns^	0.38 ^ns^	0.17 ^ns^	−0.05 ^ns^	−0.48 ^ns^	0.55 ^ns^	−0.08 ^ns^	‐
SPAD × crop injury	−0.06 ^ns^	0.12 ^ns^	0.36 ^ns^	−0.37 ^ns^	0.70 *	−0.19 ^ns^	−0.30 ^ns^	−0.76 **	‐
Crop injury × aphid counts	−0.41 ^ns^	−0.20 ^ns^	0.37 ^ns^	−0.19 ^ns^	0.14 ^ns^	0.04 ^ns^	0.27 ^ns^	−0.06 ^ns^	‐

*Note:* Herbicide abbreviations used in the table (top row) are as follows: 24D, 2,4‐d; ATZ, atrazine; DIC, dicamba; GLU, glufosinate; GLY, glyphosate; ISX, isoxaflutole; NIC, nicosulfuron; PAR, paraquat; and UTC, untreated control. The term ‘crop injury’ refers specifically to crop injury resulting from herbicide treatments. Statistical notation: *, *P* ≤ 0.05, **, *P* ≤ 0.01, ***, *P* ≤ 0.001, and ns = not significant.

## 4 DISCUSSION

The overreliance on herbicides and their indiscriminate use for weed control has raised concerns regarding broader ecological consequences. Although herbicides are primarily intended for weed control, their potential impacts on nontarget organisms such as herbivorous insects have often been overlooked. This study addressed this gap by determining whether sublethal herbicide exposure, such as that resulting from off‐target drift, alters cotton susceptibility to aphid infestation. Under the conditions evaluated in this study, herbicide treatment was the primary determinant of aphid infestation severity, whereas application rate and cotton growth stage at exposure modified the magnitude and consistency of this response. Overall, 2,4‐d, dicamba and glufosinate consistently enhanced aphid severity, whereas atrazine, paraquat, isoxaflutole and nicosulfuron maintained low infestation levels. This ranking clearly distinguishes the ‘high‐risk’ and ‘low‐risk’ herbicides based on their indirect impacts on aphid populations.

### 4.1 Herbicide as the primary driver of aphid infestation

Our findings that aphid infestation was significantly higher in cotton plots treated with 2,4‐d and dicamba provide compelling evidence that, beyond their role as weed control agents, synthetic auxins may induce morphological, physiological and biochemical changes in host plants that increase their attractiveness and susceptibility to phloem‐feeding insects.^3^, ^7^, ^10^, ^40^, ^46^, ^49^, ^50^, ^67^, ^68^ These effects are likely to be mediated through disruptions to hormonal signaling pathways such as natural auxins (e.g. IAA), JA, SA and ET, or through crosstalk among them, all of which are core regulators of plant defense.^10^, ^11^, ^15^, ^40^, ^46^, ^49^, ^50^, ^67^, ^69^, ^70^, ^71^, ^72^ Although the specific mechanisms remain unclear, interference with hormonal crosstalk may alter host nutritional composition,^73^ defense signaling, and herbivore‐induced plant volatile (HIPV) emissions,^10^, ^36^, ^46^, ^67^, ^69^, ^71^, ^72^, ^74^, ^75^ thereby disrupting multitrophic interactions.^76^ Sublethal herbicide exposure to certain herbicides may disrupt SA‐mediated defenses against piercing‐sucking insects, leading to differential aphid reproduction. Similar defense modulation has been reported for neonicotinoid insecticides, which disrupt SA‐mediated systemic acquired resistance (SAR) and increase plant susceptibility to herbivorous arthropods across multiple crops.^77^

An additional, and potentially critical, mechanism may involve herbicide‐induced changes in plant nitrogen (N) dynamics and associated trophobiotic effects. Although the overall response patterns were consistent, several herbicide treatments that supported relatively low aphid infestation in the field exhibited substantially higher aphid populations under glasshouse conditions. This discrepancy may reflect higher baseline N availability in adequately fertilized glasshouse plants compared with field‐grown plants that did not receive supplemental fertilizer. Because aphid reproduction is closely linked to phloem N availability, elevated N levels, such as those associated with fertilization or reduced plant competition, have frequently been linked to increased aphid populations through trophobiotic interactions.^78^, ^79^, ^80^ For instance, 2,4‐d‐treated rice increased plant N and larval growth,^81^ whereas insecticide exposure increased essential amino acids in cotton sap, potentially contributing to higher *A. gossypii* densities.^48^ It is therefore plausible that sublethal exposure to synthetic auxin may alter phloem N profiles, thereby enhancing aphid reproduction and contributing to the infestation patterns observed here. However, this mechanism remains speculative and requires further testing.

In aphids, dispersal and the location of new hosts by alates are primarily governed by visual cues, sometimes modulated by plant volatiles, whereas host acceptance and subsequent performance are determined largely by gustatory cues during probing and by phloem nutritional quality.^34^, ^82^ Amino acids in the phloem are the dominant but often limiting N source for aphids, and changes in their concentrations or profiles can substantially affect aphid growth, fecundity, and population development.^48^, ^78^, ^83^, ^84^, ^85^, ^86^ These findings support the hypothesis that sublethal glufosinate exposure could alter phloem amino acid composition, increasing host nutritional suitability and enhancing aphid feeding and reproduction after settling. It also has been reported to negatively affect predatory mites, potentially disrupting biological control and indirectly increasing aphid populations.^87^ Likewise, glyphosate exposure can modulate plant defense signaling and increase susceptibility to insect herbivory.^3^, ^23^, ^24^, ^39^, ^43^, ^54^ Studies showed that glyphosate‐treated plants may experience increased herbivory^54^ and altered aphid life‐history traits under sublethal exposure.^88^ Thus, these findings underscore the need to reassess the assumption of ecological neutrality in herbicide risk assessments, especially in drift‐prone environments.^7^, ^89^, ^90^

### 4.2 Contrasting aphid responses among herbicides

Our data suggest that herbicide chemistry and MoA may be stronger drivers of aphid response than dose *per se*, except in the glasshouse, where strong herbicide × dose interactions emerged. Supporting this, previous studies reported rate‐independent increases in phloem‐feeder infestations under synthetic auxin herbicide exposure,^46^, ^67^, ^91^ highlighting the role of specific MoAs. Within this context, herbicides in our study exhibited distinct response patterns, falling into groups that increased, reduced, or did not measurably affect aphid infestation. 2,4‐d, dicamba and glufosinate were consistently associated with increased aphid infestation, particularly during early crop growth stages, confirming their aphid‐enhancing effects discussed in Section 4.1. By contrast, atrazine, paraquat and nicosulfuron were associated with lower aphid infestation than in untreated controls. Isoxaflutole showed the most consistent suppressive pattern: across environments and years, it was associated with aphid infestations that were significantly lower than those of the untreated control. Glyphosate differed from the suppressive group and did not consistently differ from the untreated control.

These patterns were consistent with prior reports of neutral‐to‐minimal nontarget effects and reduced insect abundance and performance under these chemistries.^92^, ^93^, ^94^ For instance, atrazine exposure has been shown to disrupt insect development and survival across multiple species,^95^ including altered emergence timing and skewed sex ratios in *Trichogramma* species^93^ as well as toxic effects in the corn pests *Sipha maydis* and *Schizaphis graminum*.^92^ Paraquat can induce oxidative stress and mitochondrial dysfunction in insects,^96^ and may further reduce aphid suitability indirectly by causing rapid oxidative injury in plant tissues. Likewise, nicosulfuron has shown species‐specific effects on Trichogrammatidae (Hymenoptera) species, with inhibitory responses in some species and stimulatory responses in others.^97^ Although direct insect toxicity of isoxaflutole has rarely been documented, it disrupts carotenoid biosynthesis, leading to bleaching and systemic physiological changes in plants.^94^ These changes are likely to alter phloem quality or activate stress‐related plant defense and indirectly reduce host suitability for aphids. Thus, atrazine, isoxaflutole and nicosulfuron disrupt key physiological pathways, including photosynthesis, carotenoid biosynthesis and amino acid synthesis, respectively,^90^, ^92^, ^93^, ^94^, ^95^ potentially altering host quality that reduce aphid feeding and reproduction. However, the exact mechanism of aphid suppression by these herbicides remains to be investigated.

### 4.3 Crop developmental stage, herbicide injury and environmental conditions modulate aphid responses

The observed variation in aphid responses across different crop growth stages highlights stage‐dependent modulation. Our field results showed that aphid infestation was generally highest when herbicide exposure occurred during the early vegetative stage and declined as plants matured. During reproductive transition, carbon (C) and N are increasingly redirected to floral and reproductive sinks, altering phloem composition through an increased C:N ratio during floral induction^98^ and altered amino‐acid loading.^99^ These shifts have important ecological consequences because aphids rely on phloem sugar and amino‐acid profiles for growth and reproduction.^100^ This stage‐dependent variation could also reflect the modular nature of plant architecture,^101^ leading to tissue‐specific differences in herbicide uptake, sensitivity and recovery.^102^ Because synthetic auxin herbicides are often applied during early crop development,^7^, ^103^ any herbicide‐induced disruptions during this stage may have cumulative effects on plant–insect interactions, particularly because aphids respond strongly to plant architecture and volatile cues altered by herbicide exposure.^53^, ^90^ Therefore, the net outcome for pest attraction or deterrence may differ substantially depending on when and how herbicide exposure occurs.

Notably, in the 2022 field trial, only 2,4‐d and dicamba demonstrated significant positive correlations between crop injury and aphid infestation, suggesting that herbicide‐induced physiological stress may compromise plant defenses and inadvertently enhance host suitability. These findings align with previous reports that low‐dose herbicide exposure, as a form of mild abiotic stress, can stimulate aphid reproduction and host‐seeking behavior.^88^, ^103^ By contrast, atrazine and paraquat showed negative correlations, suggesting that severe stress may reduce host quality sufficiently to deter aphid colonization. Prior studies also indicate that heavily stressed or senescing plants limit aphid survival and fecundity.^103^ Glasshouse results further supported this pattern, with higher glufosinate and paraquat doses associated with reduced SPAD readings, indicating reduced host quality under extreme stress. However, it remains unclear whether reductions in chlorophyll were a direct consequence of herbicide action or secondary effects of aphid feeding. Therefore, we further hypothesize that dose‐dependent aphid responses differ between systemic and contact herbicides. Systemic herbicides may alter whole‐plant metabolism and phloem quality, potentially enhancing aphid performance under moderate stress. By contrast, contact herbicides cause localized necrosis and tissue degradation, reducing phloem accessibility and suppressing aphid establishment at higher injury levels. This interpretation aligns with our observations that moderate stress increased aphid infestation, whereas severe injury reduced.

The contrasting aphid responses between field and glasshouse conditions reflect environment‐dependent herbicide expression. Under field conditions, herbicides can dissipate rapidly through volatilization, wash‐off, plant metabolism, and dilution by new growth, while fluctuating temperature and humidity may further reduce uptake and activity.^103^, ^104^, ^105^ Several herbicides evaluated in this study act through light‐dependent pathways (e.g. paraquat, atrazine and isoxaflutole).^106^ Under the high, fluctuating, UV‐rich irradiance of the field, these herbicides are likely to have caused greater injury, reducing host quality and suppressing aphid infestation, whereas under stable glasshouse light, the same treatment is likely to have acted as a sublethal physiological stressor. This shift explains the observed reversal in aphid response with the herbicide × dose interaction under glasshouse conditions. Additionally, field infestations resulted from natural colonization under environmental and natural‐enemy pressures and were assessed as visual severity ratings, whereas glasshouse infestations originated from a controlled aphid release in the absence of predators and were quantified as aphid counts per plant. These conditions may have amplified differences in host preference and reproduction that were partially buffered under field conditions. This field–glasshouse discrepancy may also reflect volatile‐mediated cue interference. Under stagnant air in the glasshouse, volatile organic compounds (VOCs) emitted from more attractive plants may accumulate and mix with cues from neighboring plants, reducing aphid host discrimination and leading to less selective settling. Because sublethal herbicide exposure can alter VOC emissions,^15^ and neighboring plant volatiles can reshape insect behavior,^107^ these spatial patterns suggest that herbicide effects on aphids are mediated not only by plant physiology, but also by microclimate, airflow and host spatial arrangement.

### 4.4 Implications for ecology and integrated Pest management (IPM)

From an ecological perspective, herbicide‐induced changes in plant traits may disrupt multitrophic interactions and reduce the functional capacity of natural enemies. Disruptions in HIPV profiles or alterations in phloem chemistry may not only attract herbivores such as aphids, but also impair the plant's ability to recruit or retain predators and parasitoids. These disruptions, particularly under sublethal or drift exposures, could destabilize predator–prey dynamics, creating trophic mismatches that ultimately reduce the resilience of the system. In IPM systems that rely on ecological balance, herbicide‐mediated shifts can weaken biological control and increase the risk of secondary pest outbreaks. From a pest management standpoint, these findings suggest a potential concern. Herbicides, particularly when applied at sublethal doses or under drift conditions, may inadvertently influence pest dynamics by enhancing host suitability or plant susceptibility. This may prompt increased reliance on insecticides to manage resurgent aphid populations, potentially raising production costs and contributing to the development of insecticide resistance. Such outcomes are particularly challenging in systems seeking to reduce chemical inputs or those that depend heavily on biological control as a core sustainability strategy.

## 5 CONCLUSION

Our multi‐environment study provides robust empirical evidence that sublethal herbicide exposure can inadvertently influence cotton's susceptibility to aphid infestation. These effects were most pronounced when applications occurred at early vegetative stages and varied among herbicide groups, highlighting the role of crop phenology and herbicide characteristics in shaping herbicide–pest interactions. The observed correlations between plant injury, reduced chlorophyll content, and aphid severity suggest that herbicide‐induced biochemical and physiological changes alter host cues that influence herbivore behavior. Spatial analyses further indicate that herbicide drift and airflow dynamics can reinforce local pest aggregation, amplifying ecological consequences beyond treated areas. Collectively, our findings reveal that herbicides are not always ecologically neutral toward nontarget herbivores. Incorporating herbicide–insect interactions into risk assessment and IPM frameworks is therefore essential to prevent unintended pest outbreaks and safeguard the sustainability of agricultural systems. Future research should focus on elucidating the physiological and molecular mechanisms underlying these interactions, testing responses across cultivars and environments, and quantifying drift thresholds relevant to field practice. Such integrative approaches will enable the design of herbicide programs that minimize pest facilitation while maintaining effective weed control across diverse agricultural landscapes.

## CONFLICT OF INTEREST

The authors declare no conflicts of interest.

## ETHICS STATEMENT

This article does not report any studies involving human participants or animals that would require ethical approval.

## Data Availability

The data that support the findings of this study are available on request from the corresponding author. The data are not publicly available due to privacy or ethical restrictions.
